# Targeting NAMPT‐OPA1 for treatment of senile osteoporosis

**DOI:** 10.1111/acel.14400

**Published:** 2024-11-14

**Authors:** Chao‐wen Bai, Bo Tian, Ming‐chao Zhang, Qin Qin, Xin Shi, Xi Yang, Xiang Gao, Xiao‐zhong Zhou, Hua‐jian Shan, Jin‐yu Bai

**Affiliations:** ^1^ Department of Orthopedics The Second Affiliated Hospital of Soochow University Suzhou Jiangsu China; ^2^ Institution of Neuroscience Soochow University Suzhou Jiangsu China; ^3^ Suzhou Medical College Soochow University Suzhou Jiangsu China

**Keywords:** cellular senescence, mesenchymal stem cell, mitochondrial function, NAMPT, Optic atrophy protein 1

## Abstract

Senescence of bone marrow mesenchymal stem cells (BMSCs) impairs their stemness and osteogenic differentiation, which is the principal cause of senile osteoporosis (SOP). Imbalances in nicotinamide phosphoribosyltransferase (NAMPT) homeostasis have been linked to aging and various diseases. Herein, reduction of NAMPT and impaired osteogenesis were observed in BMSCs from aged human and mouse. Knockdown of *Nampt* in BMSCs promotes lipogenic differentiation and increases age‐related bone loss. Overexpression of *Nampt* ameliorates the senescence‐associated (SA) phenotypes in BMSCs derived from aged mice, as well as promoting osteogenic potential. Mechanistically, NAMPT inhibits BMSCs senescence by facilitating OPA1 expression, which is essential for mitochondrial dynamics. The defect of NAMPT reduced mitochondrial membrane potential, interfered with mitochondrial fusion，and increased SA protein and phenotypes. More importantly, we have confirmed that P7C3, the NAMPT activator, is a novel strategy for reducing SOP bone loss. P7C3 treatment significantly prevents BMSCs senescence by improving mitochondrial function through the NAMPT‐OPA1 signaling axis. Taken together, these results reveal that NAMPT is a regulator of BMSCs senescence and osteogenic differentiation. P7C3 is a novel molecule drug to prevent the pathological progression of SOP.

## Graphical abstract

This study shows that reduced nicotinamide phosphoribosyltransferase (NAMPT) levels are linked to aging‐related BMSC dysfunction. NAMPT regulates BMSC senescence by promoting OPA1 expression, crucial for mitochondrial dynamics. The NAMPT activator P7C3 enhances mitochondrial function via the NAMPT‐OPA1 axis and helps prevent SOP, highlighting its potential as a therapeutic strategy.

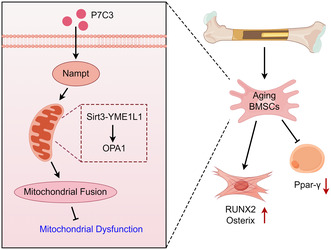

**Abbreviations**
BMD: bone mineral density
BMSCs: bone marrow mesenchymal stem cells
BV/TV: bone volume per tissue volume
IMM: inner mitochondrial membrane
NAMPT: nicotinamide phosphoribosyltransferase
OCN: osteocalcin
OPA1: Optic atrophy protein 1
SA: senescence‐associated
SOP: senile osteoporosis
Tb.N: trabecular number
Tb.Sp: trabecular spacing
Tb.Th: trabecular thickness
β‐gal: β‐galactosidase

## 1 INTRODUCTION

Senile osteoporosis (SOP) has become a worldwide bone disease due to the aging of the world population (Compston et al., 2019). SOP typically occurs in individuals over 70 years old and is an inevitable degenerative disease associated with aging. It is characterized by disruption in bone metabolism, including the deterioration of bone microarchitecture, reduction of bone trabecular, and thinning of cortical bone, which lead to decreased bone strength and increased susceptibility to fracture susceptibility (Fuggle et al., 2019). As life expectancy rises and the elderly population continuous to increase, SOP and related fractures not only increase the morbidity and mortality of elderly individuals, but also substantially exacerbate the financial burden on public health, has become a serious social problem (Vilaca et al., 2022). Decreased bone formation and increased bone marrow adiposity are considered primary causes of skeleton changes caused by aging (Li et al., 2020; Song et al., 2022).

Stem cell senescence and functional decline have emerged as core features of aging, marked by a lack of regenerative potential and loss of tissue dynamic homeostasis in various organs (Liang et al., 2021; Moiseeva et al., 2023; Zhang et al., 2016). Among these, the most characteristic examples include age‐related diseases of the skeletal system, where bone mesenchymal stem cells (BMSCs) reside and mediate age‐related osteoporosis (Guo et al., 2021; Qi et al., 2017; Wang et al., 2020; Zhang et al., 2023). BMSCs have the potential to differentiate into various cell types, including osteoblasts, chondrocytes, and adipocytes (Chen et al., 2016; Yu et al., 2023). The lineage fate determination between osteoblasts and adipocytes is mutually exclusive, age‐related osteoporosis is partly explained by the reduced ability of BMSCs from older patients to differentiate into osteoblasts and the increased ability to differentiate into adipocytes (Hu et al., 2022; Wang et al., 2022). However, the molecular mechanisms behind the transition from youthful to aging BMSCs remain elusive.

Previous studies have found that aging significantly reduces levels of the essential cofactor nicotinamide adenine dinucleotide (NAD^+^) in multiple peripheral tissues (Covarrubias et al., 2021; Gan et al., 2021). This age‐associated decrease in NAD^+^ levels is attributed to a decline in the protein levels of nicotinamide phosphoribosyltransferase (NAMPT), the rate‐limiting enzyme in salvage NAD^+^ biogenesis (Nacarelli et al., 2019). In addition, cells undergoing mitochondrial dysfunction‐associated senescence have lower NAD^+^/NADH ratios (Chini et al., 2021). NAD^+^ plays a key pathophysiological role in the development of tissue senescence (Covarrubias et al., 2021). For example, the NAMPT pathway becomes deficient in tissue aging (Stein & Imai, 2014; Yoshida et al., 2019), whereas overexpression of *Nampt* and supplementation with NAMPT have been used as prophylactic anti‐aging strategies (Frederick et al., 2016; Ratnayake et al., 2021; Yoshida et al., 2019). Nevertheless, the relationship between the NAMPT pathway and age‐related osteoporosis, along with the detailed mechanisms, remains to be fully elucidated.

Mitochondrial function declines during aging (Sun et al., 2016). Mitochondrial dysfunction is one of the hallmarks of aging, contributing to the aging process by causing bioenergetic abnormalities, enhancing ROS production, triggering unexpected permeability of mitochondrial membranes, and mediating inflammation and cell death (López‐Otin et al., 2023). Mitochondrial dysfunction is important for stem cell senescence (You et al., 2023). Studies have confirmed mitochondria of *Nampt*‐deleted cells are rounded and constricted with loss of cristae, indicating mitochondrial dysfunction (Basse et al., 2021; Lin et al., 2016). Optic atrophy protein 1 (OPA1) regulates inner mitochondrial membrane (IMM) fusion and remodeling of cristae (Nyenhuis et al., 2023). Increased mitochondrial fragmentation and decreased expression of mitochondrial fusion protein OPA1 are observed in aged or pathological conditions (Amartuvshin et al., 2020). Loss of *Opa1* alters mitochondrial structure and function, leading to a precocious senescence phenotype and premature death of muscle stem cells (Baker et al., 2022; Tezze et al., 2017). Additionally, *Opa1* deficiency leads to mitochondrial dysfunction and cell senescence in mesenchymal stem cells (MSCs) (Wang et al., 2023). However, the role and mechanism of mitochondrial dysfunction in stem cell senescence remain to be elucidated.

In this study, we investigate key proteins that regulate the senescence of BMSCs. We uncover that NAMPT promotes osteogenic differentiation and inhibits adipogenic differentiation by delaying BMSC senescence during aging. Notably, intraperitoneal injection of P7C3 stimulates trabecular bone formation and decreases bone marrow fat accumulation in aged mice by promoting NAMPT expression. Thus, our study provides a new mechanism and a novel therapeutic target for age‐related bone loss.

## 2 METHODS

### 2.1 Mice

C57BL/6 mice were purchased from the Laboratory Animal Center of Soochow University. All animal experiments were approved by the Animal Ethics Committee of Soochow University. All animal experiment operations and welfare complied with international ethical principles and national regulations. All experiments complied with relevant animal ethics regulations and had been approved by the Ethics Committee of Soochow University (SUDA20231215A04).

### 2.2 Human bone marrow MSCs

The inclusion and exclusion criteria of clinical patients are detailed Table S1. Human BMSCs were obtained from eight donors with femur fractures through bone marrow aspiration. Approximately 5–10 mL of bone marrow cells were collected into vacuum blood collection tubes containing heparin sodium. Mononuclear cells containing BMSCs were isolated at 600 g/25 min via centrifugation at room temperature using Ficoll–Hypaque (1.077 g/mL) (C0025‐200 mL, Beyotime). Cells from the milky ring layer were aspirated and washed with PBS.

### 2.3 Intra‐bone marrow injection of adeno‐associated virus

Recombinant adeno‐associated serotype 5 virus with Prrx1 promoter for *Nampt* knockdown (AAV5‐Prrx1‐*Nampt* shRNA) was purchased from Obio Technology Co. (Shanghai, China). Following a previously described protocol (Li et al., 2018), the AAV virus titer used in this study was 5 × 10^12 vector genomes/ml. Mice were injected with 5 μL of AAV5‐Prrx1‐*Nampt* shRNA and AAV5‐Prrx1‐shC into the femoral bone marrow cavity of 5‐month‐old C57B/6 mice. This led to BMSCs‐specific knockdown of *Nampt* (NAMPT‐eKD) as the viral construct contained BMSCS‐specific Prrx1 promoter. Three weeks after the intra‐bone marrow injection, femurs were collected and analyzed.

### 2.4 Aging mice model

Beginning at 17 months of age, P7C3 at 20 mg/kg or vehicle was administered daily to male mice by intraperitoneal injection. The mice were sacrificed at 18 months, following 1 month of P7C3 or vehicle treatment, and the femurs were collected for subsequent experiments.

### 2.5 Histological analyses and immunostaining

The femurs were fixed for 48 h with 4% paraformaldehyde, incubated in 10% EDTA for decalcification 2 weeks, and embedded in paraffin after dehydration. 6 μm‐thick femoral sections were made and stained with hematoxylin and eosin (H&E, Solarbio Science & Technology Co., Ltd., China) and Masson reagent (Solarbio Science & Technology Co., Ltd., China) following the manufacture's protocol. For Immunohistochemistry (IHC) stain, the sections were processed for osteocalcin (OCN) using the antibody from Abcam (ab93876) and NAMPT using the antibody from proteintech (11776‐1‐AP, 1:200). For immunostaining, the sections were processed for CD90 (Abcam, ab307736) and p21 (Santacruze, sc‐6246). Secondary antibodies were purchased from Abcam: goat anti‐rabbit Alexa 488 (Abcam, ab150077) and goat anti‐mouse Alexa 594 (Abcam, ab150116).

Images were acquired under an optical microscope (Olympus CX31, Tokyo, Japan) or a confocal microscope. The number of adipocytes per square millimeter of marrow tissue (N. AdCs/Ar/mm^2^) and the numbers of osteoblasts per millimeter of bone surface (N. OBs/BS/mm) were quantized by ImageJ.

### 2.6 μCT analysis

The mouse femurs were skinned and fixed in 70% ethanol, then subjected to μCT scanning by SkyScan 1172 high‐resolution micro‐CT scanner (SkyScan, Belgium) (Bruker, Kartuizersweg, Belgium) at a 9 μm resolution for quantitative analysis. The scanning parameters were set to a voltage of 50 kV, a current of 500 uA and a rotation step of 0.5°. The volume of interest for trabecular bone analysis, located 840 μm from the growth plate and extended a further 1680 μm longitudinally in the proximal direction, was analyzed for bone volume per tissue volume (BV/TV), trabecular number (Tb.N), trabecular thickness (Tb.Th), trabecular spacing (Tb.Sp) and bone mineral density (BMD) with μCT Tomography software (Scanco USA). For cortical bone, the region of analysis was 5% of femoral length in the femoral mid‐diaphysis for measuring cortical thickness (Ct.Th).

### 2.7 The molecular docking of P7C3 and NAMPT

The X‐ray crystal structures of NAMPT (PDB: 2GVL) were retrieved from the Protein Data Bank. The protonation state of all compounds was set at pH = 7.4, and the compounds were expanded to 3D structures using Open Babel. AutoDock Tools (ADT3) were applied to prepare and parametrize the receptor protein and ligands. The docking grid documents were generated by AutoGrid of Sitemap, and AutoDock Vina (1.2.0) was used for docking simulation. The optimal pose was selected to interaction analysis. Finally, the protein‐ligand interaction figure was generated by PyMOL.

We analyzed the interactions between protein and ligand, dentifying and classifying all functional residues according to their interactions. Multiple groups of residues formed interactions between receptor protein and ligand, such as the hydrogen bond formed by GLU376 of NAMPT and ligand. With these interaction forces, the binding energy of protein‐ligand complex was −7.2 kcal/mol, indicating excellent performance.

### 2.8 Cell differentiation

BMSCs were isolated from femurs and tibias of 4 to 6‐week‐old C57BL/6 mice. Briefly, the femurs and tibias were dissected under sterile conditions and placed in α‐MEM (SH30265.01; Hyclone, Logan, USA) with 1% Penicillin–Streptomycin (PS; P1400; Solarbio, Beijing, China). The epiphyses of femurs and tibias were removed and marrows were flushed with a 1‐mL syringe using α‐MEM + 1% PS. Isolated bone marrow cells were then plated in 10‐cm dish and cultured in medium (α‐MEM containing 10% FBS,1% P/S) for 24 h. The nonadherent cells were removed, and the adherent cells were used for BMSCs and BMSCs‐derived osteoblastic cells.

For osteoblast differentiation, BMSCs were seeded in 6‐well plates and cultured in α‐MEM containing 10% FBS, 1% penicillin/streptomycin, 0.1 μM dexamethasone, 50 μg/mL L‐ascorbic acid (Sigma, A5960), and 10 mM β‐glycerophosphate disodium salt hydrate (Sigma, G9422). The cell culture media was refreshed every 3 days, supplemented with either 10 μM P7C3 or DMSO vehicle control. For ALP staining assay, the medium was removed after 7 days, the samples were fixed in 4% PFA for 30 min, and then washed with PBS. The BCIP/NBT Alkaline Phosphatase Color Development Kit (C3206) was purchased from Beyotime Biotechnology. According to the instructions, staining working solution was prepared by mixing 10 mL ALP chromogenic buffer, 33 μL BCIP solution, and 66 μL NBT solution. Samples were incubated in the dark for 24 h. The ALP positive areas, which appeared blue, were quantified using ImageJ software. After 21 days, the formation of calcium nodules was detected by modified Alizarin Red S (ARS) method. The Calcium salt staining solution Kit (G3280) was purchased from Beijing Solarbio Science & Technology Co. Ltd. The stained calcium nodules bound ARS and appear red. Then use ImageJ software to quantify the red area, determined as stained area/total area × 100%.

For adipogenic differentiation, BMSCs were seeded in 6‐well plates and cultured in HG‐DMEM containing 10% FBS, 1% penicillin/streptomycin, 1 μM Dexamethasone, 10 μg/mL insulin, 200 μM indometacin, and 0.5 mM 3‐Isobutyl‐1‐methylxanthine (IBMX). The differentiation medium was changed every 2 days and supplemented with either 10 μM P7C3 or DMSO as a vehicle control. To detect the formation of lipid droplets, the cells were stained with Oil Red O Stain Kit (G1262; Solarbio) 15 days after adipogenic induction.

### 2.9 *Nampt* knock down or overexpress

At 100, 000 cells per well, BMSCs were seeded into six‐well tissue culture plates, and *Nampt* shRNA and *Nampt* overexpression plasmid lentivirus added. The culture medium was replaced with fresh puromycin‐containing culture medium every 2 days until resistant colonies were formed (10–12 days). In stable cells, *Nampt* knockdown was verified by Western blotting and quantitative real time PCR (qPCR).

### 2.10 JC‐1 staining

JC‐1 was used to assess the mitochondrial membrane potential changes. Briefly, BMSCs were cultured in medium containing 10 μg/ ml JC‐1 (Beyotime Biotechnology, China) at 37°C for 20 min. After washing, the cells were cultured in growth medium and examined by fluorescence microscopy (Leica, German). When the mitochondrial membrane potential is high, JC‐1 aggregates in the matrix of mitochondria to form the polymer (J‐aggregates), which produces red fluorescence. When the mitochondrial membrane potential is low, JC‐1 remains in the monomeric form, producing green fluorescence. The mitochondrial membrane potential was evaluated by calculating the ratios of red/green fluorescence intensity. Images captured from three independent experiments were processed using Image J software to measure the red and green fluorescence intensity.

### 2.11 MitoTracker staining

To assess mitochondrial morphology in MSCs, MitoTracker staining was employed. Briefly, MSCs were cultured in confocal dishes and subjected to various treatments. Upon reaching 50%–60% confluence, MSCs were washed three times and incubated for 15 min in complete culture medium containing 25 nM MitoTracker Green FM (C1048, Beyotime). Finally, cells were washed with complete culture medium and imaged using a laser confocal microscope (LSM 900, ZEISS).

### 2.12 Measurement of mitochondrial length

To quantify mitochondrial length, Image‐Pro Plus 6.0 software was utilized. For detailed procedures, confocal images were imported into Image‐Pro Plus 6.0. Standard Area of Interest (AOI) tracking was employed to assess cell morphology. To enhance fine details of the mitochondrial network, the “Sharpen” filter was applied under “Enhance” with settings adjusted to “5 × 5” and “Passes” set to 2. Subsequently, the mitochondrial network was refined to 1 pixel by setting the “Thinning” option to 22% under the “Morphological” filter. Next, the edges of the mitochondrial network were expanded to two pixels using the “2 × 2 Square” option under “Deliate” and the area occupied by labeled mitochondria was measured. Finally, mitochondrial length was determined by dividing the measured area by two, the width of the labeled mitochondria.

### 2.13 Senescence associated β galactosidase staining

β‐galactosidase staining was conducted following the instructions from the senescence‐associated (SA) β‐galactosidase staining kit (C0602, Beyotime Biotechnology). Briefly, the cells were fixed with 4% paraformaldehyde for 20 min. After washing three times with PBS, the dyeing working solution were added, and the cells were incubated for 12 h at 37°C. The cells were then observed and imaged under a microscope Leica (DMi8).

### 2.14 Coimmunoprecipitation

We assessed the acetylation level of YME1L1 and the interaction between Sirt3 and YME1L1 using coimmunoprecipitation (co‐IP). Proteins were extracted from BMSCs following appropriate treatment. They were coincubated overnight at 4°C with specified primary antibodies (anti‐acetylated lysine or Sirt3) and Protein A + G agarose beads (Beyotime, China). After incubation, the beads were centrifuged and washed three times, followed by elution of immunocomplexes in elution buffer. Proteins were separated by SDS‐PAGE, and membranes were subsequently incubated overnight at 4°C with the specified primary antibody (YME1L1 1:1000). The remaining steps followed a standard protocol for protein blotting.

### 2.15 Real time RT‐PCR analysis

Total RNA was extracted with TRIzol (Invitrogen, Carlsbad, CA) and reverse transcribed into cDNA using the PrimeScript RT Reagent Kit (vazyme, Nanjing, China). mRNA levels of interest genes were measured by real time qPCR (ABI‐7600 Prism equipment) using SYBR Green Master Mix (vazyme, Nanjing, China). mRNA expression of targeted genes was quantified via the ΔΔCt protocol. The nucleotide sequence of primers used in this study are listed in Table S1.

### 2.16 Western blot analyses

Total protein was extracted from cell pellets using 1 × RIPA buffer supplemented with protease and phosphatase inhibitors. Samples were incubated on ice for 30 min and then centrifuged at 12,000 g for 15 min at 4°C. Supernatants were collected and concentrations of protein samples were measured using the BCA Protein Assay Kit (Thermo Fisher, Shanghai, China). Supernatants were mixed with sample buffer, heated at 95°C for 10 min, separated on 10% SDS‐PAGE gels by electrophoresis, and subsequently electrical blotted to polyvinylidenedifluoride membranes (Merck, Germany). After being incubated with 10% skimmed milk in 1 × PBST for 1 h, proteins were revealed by primary antibodies at 4°C overnight. After washing the membrane three times with PBST, they were incubated with secondary antibodies. The blots were then subjected to chemiluminescent reagent detection according to the manufacturer's instructions. Quantification of the band intensity was performed using ImageJ software.

### 2.17 RNA sequencing

BMSCs were cultured in six‐well plates and treated with P7C3 for 1 h. Following treatment, the supernatant was carefully removed, and the cells were washed three times with 2 mL PBS. TRIzol reagent (1 mL) was then added to each well to lyse the cells, and the resulting lysate was transferred to a 1.5 mL Eppendorf tube. Total RNA was extracted using an RNA extraction kit, and residual DNA was digested using DNase (Thermo Fisher). Eukaryotic mRNA was enriched using Oligo (dT) magnetic beads, and mRNA was fragmented into shorter segments using an interrupting reagent. These mRNA fragments served as templates for the subsequent steps. Single‐stranded cDNA was synthesized using random hexamer primers, followed by the preparation of a reaction system for synthesizing double‐stranded cDNA. The resulting double‐stranded cDNA was then purified and repaired. For RNA sequencing, total RNA from the harvested cells was further assessed using an Agilent Bioanalyzer (Agilent, Waldbronn, Germany). RNA libraries were constructed using the NEB Next® Ultra II RNA Library Prep Kit for Illumina and subsequently sequenced on an Illumina HiSeq 4000 platform (Illumina, San Diego, CA, USA). Low‐quality reads and adapter sequences were filtered out from the raw data, and the remaining reads were aligned to the mouse genome (GRCm38.91). Differentially expressed genes between the treatment and control groups were identified using DESeq2 v1.20 in R v3.5.1. Genes with expression changes greater than 1.3‐fold and *p* < 0.05 were considered significantly different. The identified genes were further analyzed, including Kyoto Encyclopedia of Genes and Genomes (KEGG) pathway enrichment analysis.

### 2.18 Reagents

Primary antibodies against p53 (sc‐126), p21 (sc‐6246), p16 (sc‐1661) were purchased from Santa Cruz Biotech. Primary antibodies against RUNX2 (12556) were purchased from Cell Signaling Technology. Primary antibodies against Pparγ (16643‐1‐AP) and NAMPT (11776‐1‐AP) were purchased from Proteintech. Primary antibodies against Osterix (ab209484) and CD90 (ab307736) were purchased from abcam. NAMPT (PA1‐1045) was purchased from Thermo Fisher. Nonspecific antibodies have not been used.

### 2.19 Statistical analysis

The Shapiro–Wilk test was used to evaluate the normality of the numerical data. All quantitative data were presented as mean ± SD, based on a minimum of three independent experiments. The two‐tailed unpaired *t* test was used to compare statistical differences between two groups. One‐way analysis of variance (ANOVA) plus Tukey's post hoc test were used for multiple groups. A *p*‐value of less than 0.05 was considered statistically significant in comparison to the control groups.

## 3 RESULTS

### 3.1 The expression of NAMPT in the bone tissue of age‐related osteoporosis was abnormally reduced

**FIGURE 1:**
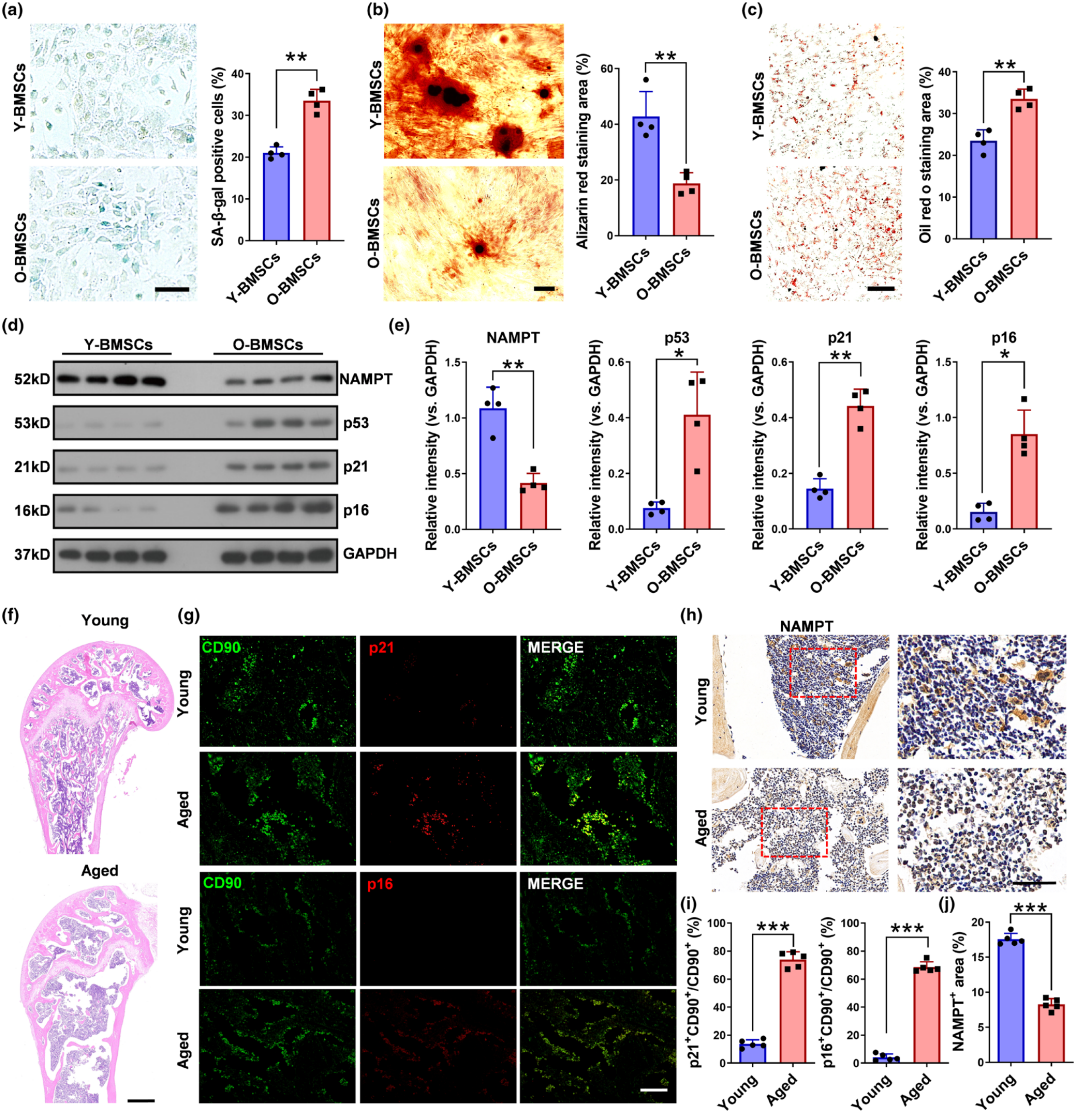
NAMPT was decreased in aged bone tissues. (a) Representative images of β‐Gal staining and quantification of β‐Gal‐positive cells of BMSCs isolated from young and aged fracture patients. *n* = 4 per group. Scale bar: 50 μm. (b) Representative images of Alizarin Red staining under osteogenic induction and quantification of calcification of BMSCs isolated from patients. *n* = 4 per group. Scale bar: 50 μm. (c) Representative images of Oil Red O staining under lipogenic induction and quantification of the percentages of Oil Red O + (red) areas isolated from patients. *n* = 4 per group. Scale bar: 50 μm. (d, e) Western blot analysis of the relative levels of NAMPT, p53, p21, and p16 proteins in BMSCs from young and aged fracture patients. *n* = 4 per group. (f) Representative images of H&E staining of femur sections from young and aged mice. *n* = 5 per group. Scale bar: 500 μm. (g) Representative images of CD90/p21 and CD90/p16 immunofluorescence staining in femora form young and aged mice. *n* = 5 per group. Scale bar: 50 μm. (h) Representative images of NAMPT immunohistochemical staining in femora form young and aged mice. *n* = 5 per group. Scale bar: 50 μm. (i) Quantification of p21^+^CD90^+^ BMSCs or p16^+^CD90^+^ BMSCs relative to CD90^+^ BMSCs in femur marrow. (j) Quantification of the percentage of NAMPT+ areas in femur marrow. Data were presented as mean ± SD. *p < 0.05, **p < 0.01, by Student's *t* test.

The decline in bone formation associated with aging is closely linked to the impairment of BMSCs. To investigate the expression of NAMPT in age‐related osteoporosis, BMSCs were extracted from four young fracture patients with normal bone density and four aged fracture patients with SOP. Compared to BMSCs from younger patients, BMSCs from older fracture patients exhibited significantly higher levels of senescence, as indicated by β‐Galactosidase (β‐Gal) staining (Figure 1a). Additionally, older patients demonstrated a lower osteogenic differentiation potential (Figure 1b), and higher adipogenic differentiation ability (Figure 1c). Western blotting analysis confirmed that NAMPT was decreased, and age‐related markers were elevated in BMSCs of older patients compared to young fracture patients (Figure 1d,e).

Microcomputed tomography (μ‐CT) scans were conducted on the femur metaphysis of 6‐month‐old and 18‐month‐old mice (Figure S1a). The μCT analysis revealed that aged mice exhibited reduced BMD, trabecular bone volume/tissue volume (BV/TV), trabecular numbers (Tb.N), and trabecular thickness (Tb.Th), and increased trabecular separation (Tb.Sp). HE staining showed that aged mice femurs had a significantly fewer trabecular bone compared with those of young group (Figure 1f). We confirmed the higher expression level of p21 and p16 in BMSCs of 18‐month‐old mice relative to 6‐month‐old mice using immunofluorescence staining(Figure 1g,i), meanwhile, western blotting results showed that p53, p21 and p16 in BMSCs was elevated in 18‐month old mice (Figure S1b). Reduced NAMPT expression has been reported to induce senescence in primary stem cells (Stein & Imai, 2014). Immunohistochemical staining for NAMPT revealed that it was broadly expressed in the bone tissues of 6‐month‐old mice. However, the expression of NAMPT in the bone tissues of 18‐months‐old mice was barely detectable (Figure 1h,j).

### 3.2 NAMPT promoted osteogenesis and suppressed adipogenesis of BMSCs

To investigate the role of NAMPT in the osteogenesis and adipogenesis of BMSCs, young BMSCs were infected with lentiviral constructs expressing shRNA targeting *Nampt* or a scrambled shRNA, while aged BMSCs were infected with lentiviral constructs *Nampt* overexpression plasmid(OE‐NAMPT) or an empty vector. Then, stable cell lines were established by puromycin selection. We found that viral transfection effectively reduced NAMPT expression in young BMSCs and increased NAMPT expression in aged BMSCs. (Figure S1c–e). Osteogenic differentiation and mineralization of BMSCs were measured by ALP staining and ARS. Compared to the shC group, results showed that the osteogenic differentiation and mineralization of young BMSCs treated with osteogenic differentiation medium were decreased when *Nampt* were silenced. Conversely, overexpression of *Nampt* could promote osteogenic differentiation and mineralization of aged BMSCs (Figure S2a,b). Western blotting analysis confirmed that knockdown of *Nampt* reduced the expression of osteogenesis‐related proteins(Runx2 and Osterix)in young BMSCs, while overexpression of *Nampt* increased the expression of osteogenesis‐related proteins (Figure S2c,d). The results of qRT‐PCR analysis were consistent with the western blot findings (Figure S1f).

Another phenotype of aging in BMSCs is increased lipogenic differentiation; therefore, we verified the effect of NAMPT on lipogenic differentiation. Oil Red O staining showed that infection with sh‐NAMPT significantly increased lipid droplet formation of young BMSCs, while infection with OE‐NAMPT significantly reduced lipid droplet formation of aged BMSCs (Figure S2e,f). Similarly, knockdown of *Nampt* promoted the expression of adipogenic molecular marker Pparγ in young BMSCs, whereas overexpression of *Nampt* could reduce the expression of adipogenic molecular marker Pparγ in aged BMSCs, according to the results of qRT‐PCR analysis and western blotting (Figure S2g–i).

### 3.3 The essential roles of NAMPT in reverse BMSC senescence

**FIGURE 2:**
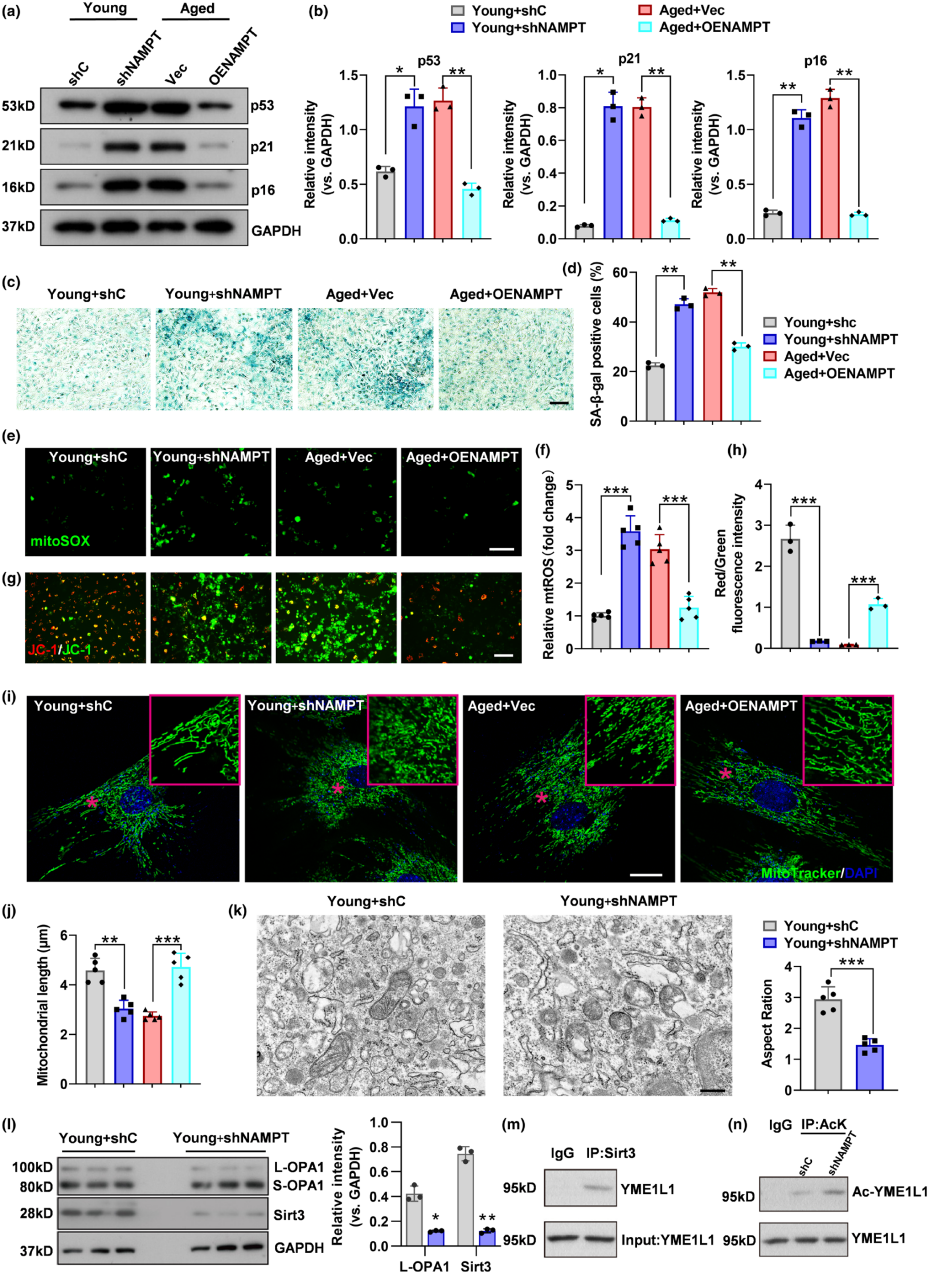
NAMPT knockdown promoted BMSCs senescence, whereas NAMPT overexpression inhibited it. (a, b) Western blot analysis of the relative levels of senescence markers p53, p21, and p16 proteins in shC, sh‐NAMPT, Vec and OE‐NAMPT BMSCs. *n* = 3 per group. (c, d) Representative images of β‐galactosidase staining and quantification of the number of β‐Gal+ BMSCs. *n* = 3 per group. Scale bar: 100 μm. (e, f) Representative Mito‐sox staining images and quantitative analysis of green fluorescence. *n* = 5 per group. Scale bar: 100 μm. (g, h) Representative images of JC‐1 staining and quantification of red/green fluorescence intensity. *n* = 3 per group. Scale bar: 100 μm. (i, j) Representative MitoTracker staining images and quantitative analysis of mitochondrial length. *n* = 5 per group. Scale bar: 10 μm. (k) Representative images of mitochondria in BMSCs from each group were observed using electron microscopy, and the aspect ratio of mitochondria was semi‐quantitatively evaluated. *n* = 5 per group. Scale bar: 2 μm. (l) Representative immunoblotting to assess OPA1 and Sirt3 levels from BMSCs infected with shC and sh‐NAMPT lentivirus. *n* = 3 per group. (m) Lysates from BMSCs were immunoprecipitated with anti Sirt3 antibody and blotted with anti‐YME1L1 antibodies. *n* = 3 per group. (n) YME1L1 acetylation level in BMSCs infected with shC and sh‐NAMPT lentivirus. *n* = 3 per group. Data were presented as mean ± SD. *p < 0.05, **p < 0.01, ***p < 0.001, by one‐way ANOVA plus Tukey's post hoc test.

Senescent BMSCs predominantly differentiate into adipocytes rather than osteoblasts. Given the anti‐lipogenic and pro‐osteogenic roles of NAMPT in BMSCs, we investigated the potential effect of NAMPT on BMSC senescence. Using western blotting assay, we found that the expression of senescence markers, including p53, p16, and p21 proteins, was significantly increased in young BMSCs with *Nampt* shRNA but dramatically decreased in aged BMSCs with *Nampt* overexpression (Figure 2a,b). Another marker of cellular senescence is the β‐galactosidase (SA‐β‐Gal). β‐galactosidase staining indicated that *Nampt* knockdown increased the number of SA‐β‐gal‐positive cells in young BMSCs, whereas *Nampt* overexpression decreased that in aged BMSCs (Figure 2c,d).

Mitochondrial dysfunction is a hallmark of cellular senescence and organ aging (López‐Otin et al., 2023). As mitochondria derived reactive oxygen species (mtROS) resulting from disturbance of mitochondrial dynamics can cause cellular senescence through DNA damage and other pathway, mtROS levels were measured in BMSCs using MitoSOX staining. The results indicated higher levels of mtROS in the *Nampt* knockdown group than in the control group, while *Nampt* overexpression reduced levels of mtROS in aged BMSCs (Figure 2e,f). Since the mitochondrial membrane potential often decreased in senescent cells and that is associated with mitochondrial dysfunction during senescence, we detected the mitochondrial membrane potential by JC‐1 staining (Figure 2g,h). Our data showed that *Nampt* knockdown resulted in a decrease of the mitochondrial membrane potential in young BMSCs, while *Nampt* overexpression caused the opposite phenotype in aged BMSCs.

The high‐resolution mitochondrial fluorescence images clearly showed that *Nampt* knockdown on young BMSCs turned mitochondria into shorter mitochondrial morphologies, whereas *Nampt* overexpression in aged BMSCs led to longer mitochondrial morphologies (Figure 2i,j). Ultrastructural analysis of confirmed the presence of smaller mitochondria in *Nampt*‐knockdown MSCs (Figure 2k).

OPA1 mediates fusion, remodeling of cristae, mitochondrial DNA maintenance, and potentially fission in the IMM. Conditional and inducible *Opa1* deletion alters mitochondrial morphology and function, leading to senescence. (Baker et al., 2022; Tezze et al., 2017). Long‐form OPA1 (L‐OPA1), located in the inner membrane, promotes mitochondrial inner membrane fusion. L‐OPA1 is cleaved at two different sites into short‐form OPA1 (S‐OPA1), which is released into the intermembrane space by two proteins YME1L1 and OMA1 (Hartmann et al., 2016). While S‐OPA1 is dispensable for fusion, its accumulation promotes mitochondrial fission. Previous studies have confirmed that the mitochondrial protein NAD^+^‐dependent deacetylase sirtuin 3 (Sirt3) deacetylates YME1L1, thereby inhibiting OPA1 processing and promoting mitochondrial fusion (He et al., 2021). We found that Sirt3 and L‐OPA1 levels were lower in *Nampt*‐knockdown MSCs compared to MSCs in the shC group, which indicated that the NAMPT‐Sirt3 axis suppresses OPA1 cleavage (Figure 2l). Sirt3 interacts with YME1L1 (Figure 2m), and *Nampt* knockdown facilitated YME1L1 acetylation compared to MSCs in the shC group (Figure 2n). These data indicate that NAMPT increases the expression of Sirt3, inhibits YME1L1 acetylation, thereby suppressing OPA1 processing and promoting mitochondrial fusion.

### 3.4 BMSCs knockdown of *Nampt* attenuated bone formation and promoted fat accumulation in young mice

**FIGURE 3:**
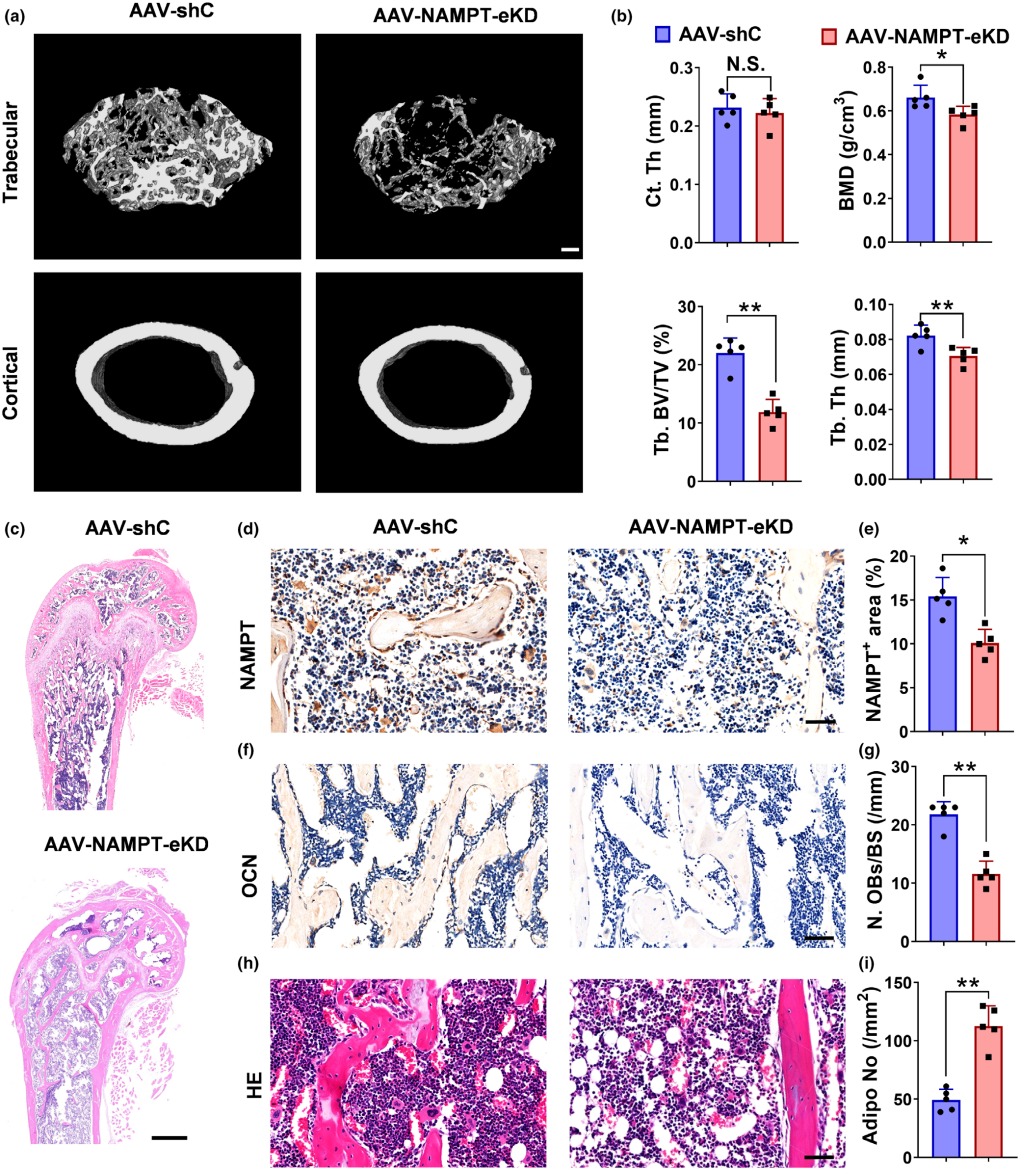
BMSCs knockdown of NAMPT attenuated bone formation fat accumulation and promoted fat accumulation in aged mice. (a, b) Representative μCT images and quantitative μCT analysis of cortical and trabecular bone microarchitecture in femora from 5‐month‐old mice with AAV5‐Prrx1‐*Nampt* shRNA or AAV‐shC transfection (BV/TV, bone volume per tissue volume; Tb.N, trabecular number; Tb.Th, trabecular thickness). *n* = 5 per group. Scale bar: 200 μm. (c) Representative images of H&E staining in femora. *n* = 5 per group. Scale bar: 500 μm. (d, e) Representative images of NAMPT immunohistochemical images and quantification of the percentage of NAMPT+ areas in femur marrow. *n* = 5 per group. Scale bar: 50 μm. (f, g) Representative images of OCN immunohistochemical staining in femora and quantification of OCN positive cells in bone surface. *n* = 5 per group. Scale bar: 50 μm. (h, i) Representative images of H&E staining in femora quantification of the number of adipocytes related to the tissue area. *n* = 5 per group. Scale bar: 50 μm. Data were presented as mean ± SD. *p < 0.05, **p < 0.01, ***p < 0.001, by Student's *t* test.

To investigate whether knockdown of *Nampt* expression could aggravate bone loss by promoting BMSCs senescence in vivo, we constructed an AAV serotype 5 with a Prrx1 promoter (AAV5‐Prrx1‐*Nampt* shRNA) to knockdown *Nampt* in BMSCs. We treated 5‐month‐old mice with AAV5‐Prrx1‐*Nampt* shRNA via intra‐bone marrow injection. One month later, the expressions of NAMPT decreased significantly in the BMSCs of the NAMPT‐eKD mice (Figure S1h,i). In addition, μ‐CT scan analysis of the femur revealed that the reduction of BMD, BV/TV and Tb.Th in NAMPT‐eKD mice group compare to AAV‐shC group (Figure 3a,b). Meanwhile, HE staining revealed that AAV5‐Prrx1‐*Nampt* shRNA‐treated femurs had significantly fewer trabecular bone compared with those of AAV‐shC group (Figure 3c). Immunohistochemical staining for NAMPT revealed that its expression was lower in femur sections of NAMPT‐eKD mice group than that of AAV‐shC group (Figure 3d,e). Furthermore, AAV5‐Prrx1‐*Nampt* shRNA‐treated femurs had significantly fewer osteoblasts on the trabecular bone surfaces (Figure 3f,g) and more bone marrow fat accumulation (Figure 3h,i).

These results suggest that knockdown of *Nampt* expression aggravates age‐related bone loss and bone marrow fat accumulation in young mice.

### 3.5 P7C3 altered gene networks and the signal transduction of BMSCs

The structure and binding mode of P7C3 and NAMPT showed that multiple groups of residues forming interactions between NAMPT and P7C3 (Figure S3a), such as the hydrogen bond formed by Glu376 and Ser379 of NAMPT and ligand (Figure S3b). These interaction forces resulted in a binding energy of −7.2 kcal/mol for the protein‐ligand complex, indicating excellent performance.

To understand the mechanistic role of P7C3 in BMSCs, we performed an in‐depth analysis of its effect on the genomic transcriptional network of BMSCs. Whole‐transcriptome RNA sequencing (RNA‐seq) was performed on BMSCs stimulated with P7C3 compared with control BMSCs. Differential expression analysis and volcano plot revealed that P7C3 significantly upregulated 298 genes and downregulated 301 genes (Figure S3c). Moreover, KEGG pathway analysis and reactome annotations of the RNA‐seq data demonstrated that P7C3 played a very critical role in aging and lipid metabolism signal transduction in BMSCs (Figure S3). The KEGG enrichment analysis of differentially expressed genes in signal transduction indicated that many signaling pathways, including calcium, PI3K‐Akt, and MAPK pathways, were regulated by P7C3 (Figure S3f). These pathways played crucial roles in osteogenic differentiation process (Chen, Shi, et al., 2023; Wu et al., 2016).

### 3.6 The NAMPT agonist P7C3 promoted osteoblast formation and inhibited adipocyte formation in BMSCs

**FIGURE 4:**
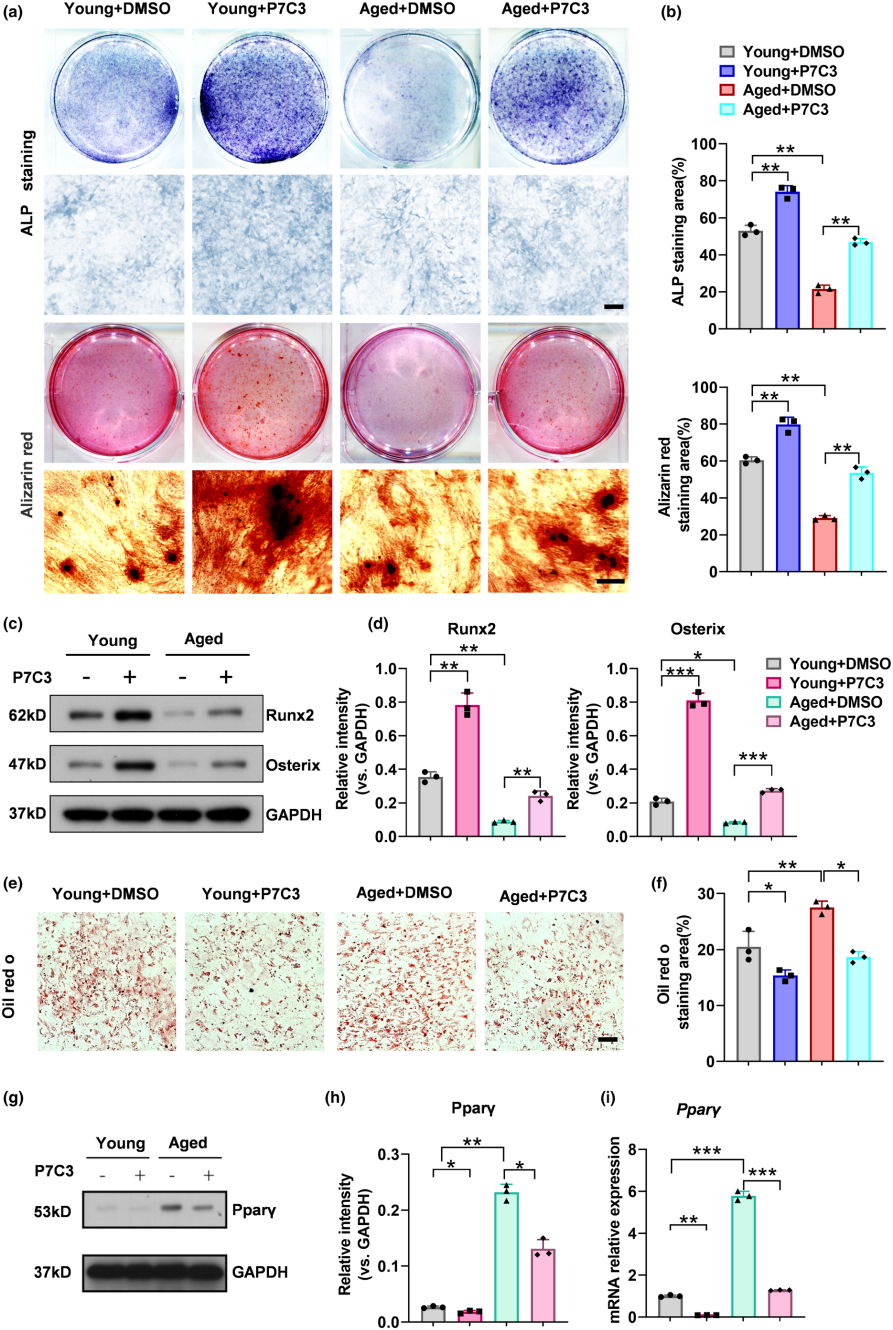
P7C3 stimulated osteogenetic differentiation of aged BMSCs. (a, b) ALP staining (a, top) and Alizarin Red staining in young and aged BMSCs treated with solvent or P7C3 under osteogenic induction and quantification of the percentages of ALP+ (blue) and ARS+ (red) areas (b). *n* = 3 per group. Scale bar: 50 μm. (c, d) Western blot analysis of the relative levels of Runx2 and Osterix in young and aged BMSCs treated with solvent or P7C3 under osteogenic induction. *n* = 3 per group. (e–i) Oil red O staining images, Western blot (g, h) and qRT‐PCR (i) analysis of Pparγ expression in young and aged BMSCs treated with solvent or P7C3 under adipogenic induction (e) and quantification of the percentage of ORO+ (red) areas. (f) *n* = 3 per group. Scale bar: 50 μm. Data were presented as mean ± SD. *p < 0.05, **p < 0.01, ***p < 0.001, by one‐way ANOVA plus Tukey's post hoc test.

To further validate our findings that NAMPT could promote osteoblasts formation and inhibit adipocytes formation, we used the specific NAMPT agonist P7C3 to test the effects of NAMPT enzymatic activity on osteogenic and lipogenic differentiation in BMSCs. ALP staining and ARS staining, respectively, showed that 1 μM P7C3 significantly promoted osteogenic differentiation and calcium nodule formation in both young and aged BMSCs under osteogenic induction (Figure 4a,b). Additionally, P7C3 profoundly augmented the expression of osteogenesis‐related genes (*Alpl, Runx2* and *Sp7*) and osteogenesis‐related proteins(Runx2 and Osterix)in young and aged BMSCs under osteogenic induction, as shown by qRT‐PCR analysis (Figure S4a) and western blotting assay results (Figure 4c,d). In contrast, P7C3 markedly reduced lipid droplet formation of young and aged BMSCs under adipogenic differentiation, as shown by Oil Red O (ORO) staining (Figure 4e,f). The changes in the mRNA and protein levels of the adipogenic molecular marker Pparγ were consistent with the observed phenotype (Figure 4g–i).

### 3.7 The NAMPT agonist P7C3 protected BMSCs against senescence by reducing the expression of senescence markers and improving the mitochondrial morphology and function

**FIGURE 5:**
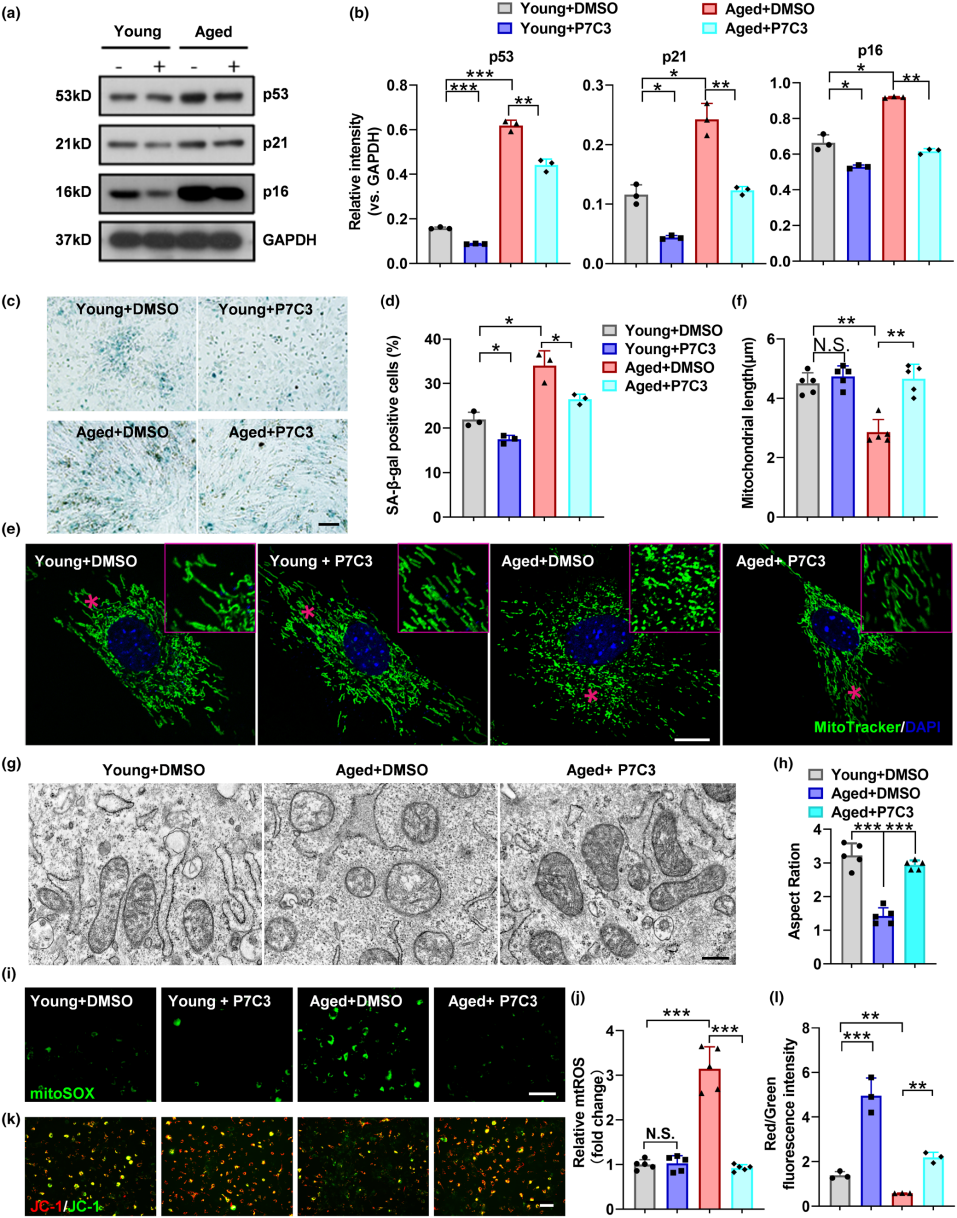
P7C3 increases mitochondrial membrane potential to prevent BMSCs senescence through NAMPT‐mediated p21/p16 downregulation. (a, b) Western blot analysis of the relative levels of p53, p21, and p16 proteins in young and aged BMSCs treated with P7C3. *n* = 3 per group. (c, d) Representative images of β‐galactosidase staining and quantification of the number of SA‐β‐Gal+ (blue) BMSCs. *n* = 3 per group. Scale bar: 100 μm. (e, f) Representative Mito Tracker staining images and quantitative analysis of mitochondrial length. Scale bar: 10 μm. *n* = 5 per group. Scale bar: 10 μm. (g, h) Representative images of mitochondria in BMSCs from each group were observed using electron microscopy, and the aspect ratio of mitochondria was semi‐quantitatively evaluated. *n* = 5 per group. Scale bar: 2 μm. (i, j) Representative Mito‐sox staining images and quantitative analysis of green fluorescence. *n* = 5 per group. Scale bar: 100 μm. (k, l) Representative images of JC‐1 staining and quantification of red/green fluorescence intensity. *n* = 3 per group. Scale bar: 100 μm. Data were presented as mean ± SD. *p < 0.05, **p < 0.01, ***p < 0.001, by one‐way ANOVA plus Tukey's post hoc test.

To further validate the anti‐senescence roles of NAMPT in BMSCs, we investigated whether the NAMPT agonist P7C3 could reverse the senescent phenotype of BMSCs. As shown by western blotting assay, P7C3 markedly suppressed the expression of p53, p21, and p16 proteins in both young and aged BMSCs (Figure 5a,b). Similarly, β‐galactosidase staining revealed that P7C3 reduced the number of SA‐β‐gal‐positive cells (Figure 5c,d). Analyses of mitochondrial morphology at the ultrastructural level confirmed the presence of shorter and smaller mitochondria in senescent BMSCs. Following treatment with P7C3, the length of mitochondria in senescent BMSCs increased, suggesting P7C3 promoted mitochondrial fusion of senescent BMSCs (Figure 5e–h). Further investigation into mitochondrial function revealed that MitoSOX staining showed that mtROS were drastically elevated due to aging, which were decreased by P7C3 treatment (Figure 5i,j). Additionally, the mitochondrial membrane potential was obviously elevated in both young and aged BMSCs treated with P7C3 (Figure 5k,l).

### 3.8 P7C3 for the treatment of SOP

**FIGURE 6:**
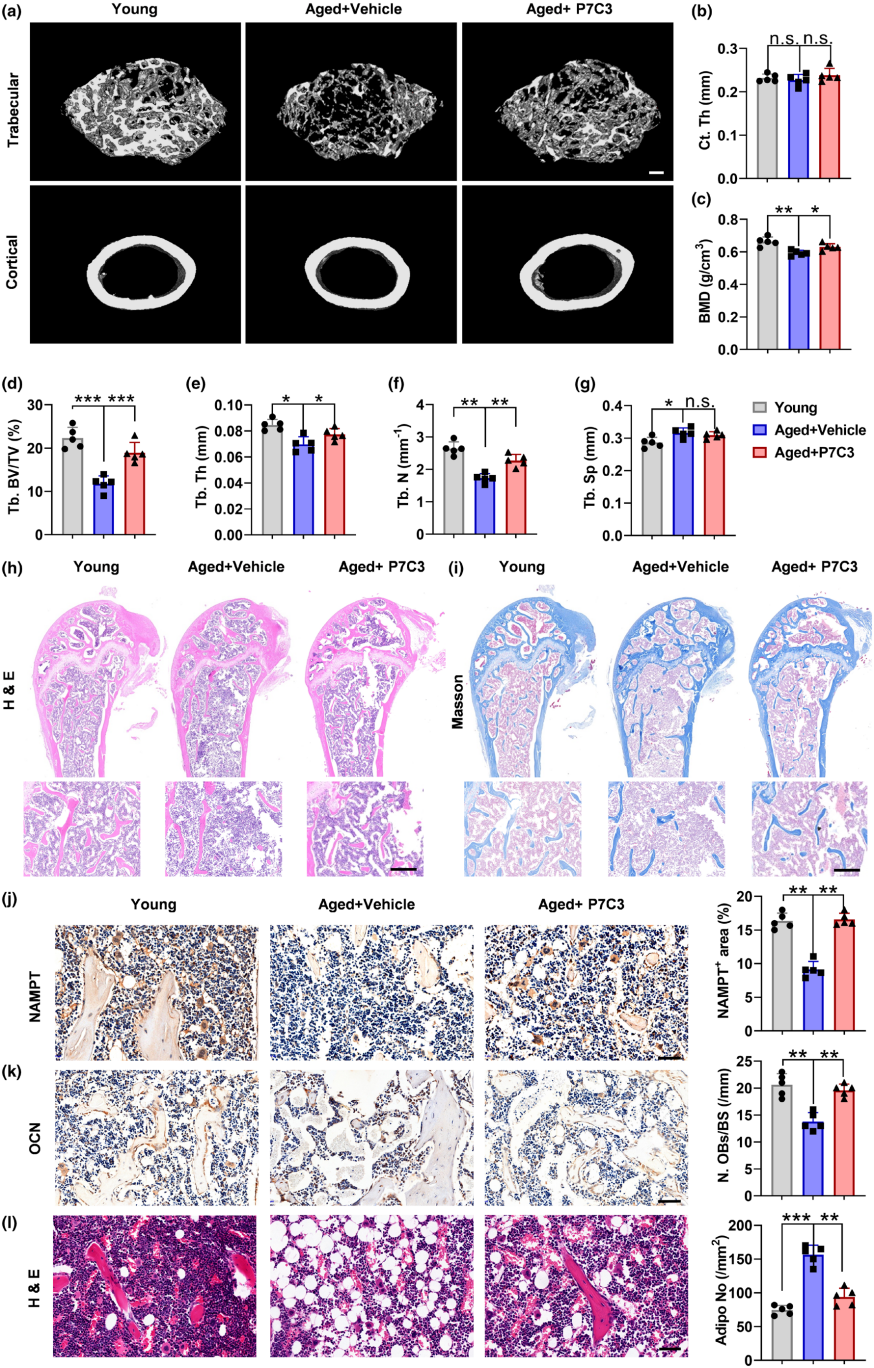
P7C3 for the treatment of aging‐related bone loss in mice. (a–g) Representative μCT images with quantitative μCT analysis of cortical and trabecular bone microarchitecture in femurs from young mice and vehicle or P7C3‐treated aged mice. *n* = 5 per group. Ct. Th, cortical thickness; BMD, bone mineral density; Tb.BV/TV, trabecular bone volume per tissue volume; Tb.Th, trabecular thickness; Tb.N, trabecular number; Tb.Sp, trabecular spacing. *n* = 5 per group. Scale bar: 200 μm. (h, i) Representative images of H&E staining and Masson staining of femur sections from young mice and vehicle or P7C3‐treated aged mice. *n* = 5 per group. Scale bar: 250 μm. (j) Representative images of NAMPT immunostaining images and quantification of the percentage of NAMPT+ areas in femur marrow from young mice and vehicle or P7C3‐treated aged mice. *n* = 5 per group. Scale bar: 50 μm. (k) Representative images and quantification of osteocalcin (OCN)‐positive cells in femur marrow. *n* = 5 per group. Scale bar: 50 μm. (l) H&E staining images and quantification of the number of adipocytes in bone marrows. *n* = 5 per group. Scale bar: 50 μm. Data were presented as mean ± SD. *p < 0.05, **p < 0.01, ***p < 0.001, by one‐way ANOVA plus Tukey's post hoc test.

Since promoting NAMPT expression in vitro with P7C3 prevented BMSCs senescence and thus promoted osteogenesis inhibiting lipogenesis, we thereby explored whether P7C3 has a protective effect against age‐related osteoporosis (Figure S4b). Stress detection was performed on the middle part of mouse femur. Statistical analysis showed that the maximum focre tolerance (Figure S4c) and hardness of femur (Figure S4d) in aged mice was significantly lower than that in young mice. However, there is no difference in the degree of deformation under the strongest mechanical load (Figure S4e). Interestingly, P7C3 ameliorated these adverse effects. Quantitative μCT analyses revealed that BMD, BV/TV, Tb.N and Tb.Th were markedly reduced, whereas Tb.Sp was increased (Figure 6a–g) in aged mice compared to young mice. Notably, these age‐induced changes were significantly diminished in the P7C3 group. To confirm the above μCT results, we performed histological analyses on femur sections. H&E and Masson staining showed that extensive bone loss occurred in aged mice. More importantly, these osteoporotic changes were obviously reduced upon P7C3 treatment (Figure 6h,i). Immunohistochemical staining for NAMPT revealed that its expression was lower in femur sections of aged mice that of young mice, however, this trend was reversed after P7C3 treatment (Figure 6j). The activity of NAMPT from bone marrow tissue was determined. The results revealed that aging significantly reduces Nampt activity, while the P7C3 increases Nampt activity (Figure S4f). Similarly, IHC showed that senescence profoundly inhibited osteogenic differentiation and promoted lipogenic differentiation, as evidenced by a large number of adipocytes and small number of OCN‐positive osteoblast mice in the femur tissue of aged mice (Figure 6k,l). However, significantly more osteoblasts and fewer adipocytes were formed in P7C3‐treated mice than in aged mice. These results indicate that activation of NMAPT by P7C3 can treat osteoporosis caused by aging though reversing the senescence of BMSCs, promoting osteogenesis and inhibiting lipogenesis.

In summary, we demonstrated that NAPMT could restrain the senescence of BMSCs and promote osteogenic differentiation by regulating p53, p21 and p16. The decreased expression level of NAPMT during aging contributes to the senescence of BMSCs, including increased adipogenesis and reduced osteogenesis. Moreover, we identified a NAMPT activator, P7C3, which could attenuate age‐related bone loss.

## 4 DISCUSSION

SA phenotypes were increased in BMSCs of SOP. NAMPT has been gaining momentum, providing critical insights into the pathogenesis of age‐associated functional decline and diseases (Yoshino et al., 2018). Our data demonstrate that NAMPT is reduced in bone tissue of SOP, facilitating SA phenotypes and inhibited osteogenic function of BMSCs. Decreased NAMPT in BMSCs disturbs OPA1 expression and mitochondrial fusion, which explains the increased SA phenotypes and dysfunction of BMSCs. Importantly, we have identified a promising therapeutic approach for mitigating bone loss caused by aging. P7C3 has been found to bind NAMPT and enhances its activity (Wang, Han, et al., 2014). Our data show that P7C3, which dependent on the NAMPT‐OPA1 signaling, effectively enhances mitochondrial function, inhibit SA phenotypes of BMSCs, and thereby delaying bone loss caused by aging. In summary, our findings highlight the importance of NAMPT‐OPA1 axis in ameliorating age‐related bone loss and offer novel therapeutic therapies.

Cellular NAD^+^ levels significantly affect the activity of enzymes that play important roles in aging defense (Covarrubias et al., 2021). Previous studies have confirmed that NAD^+^ levels decline with biological aging, and the elevation of NAD^+^ levels by genetic or pharmacologic means can counteract senescence (Covarrubias et al., 2021; Gong et al., 2022). NAD^+^ synthesis is regulated by salvage pathways which is sensitive to metabolic changes and impact several physical and pathogenic processes. NAMPT is identified as one of the rate‐limiting enzymes of this pathway (Gong et al., 2022). Reduced levels of NAMPT and NAD^+^ have been detected in peripheral tissue of old mice, such as the pancreas, white adipose tissue and skeletal muscle (Garten et al., 2015). Previous studies revealed that NAMPT in mesenchymal cells is indispensable for skeletal development. Loss of *Nampt* in mesenchymal progenitors impairs skeletal development (Warren et al., 2023). Moreover, research has confirmed that NAMPT can regulate bone metabolism by inhibiting osteoclast activity (Hassan et al., 2018). We found NAMPT expression and activity decrease while SA phenotypes increase in BMSCs with aging. Overexpression of *Nampt* decreases cellular p16, p21, p53 content and consequently enhances osteogenesis of BMSCs instead of lipogenesis in vitro. Although we did not construct *Nampt*‐conditioned knockout mice in MSCs, we clarified advantages of NAMPT in promoting bone formation using NAMPT‐eKD mice, constructed by injecting AAV serotype 5 with a Prrx1 promoter. This illustrate that NAMPT is indispensable not only for bone development but also for the prevention of SOP. NAMPT is involved in the function of inflammation‐related cells, osteoclasts and vascular endothelial cells (Travelli et al., 2018; Wang, du, et al., 2014). The influences of NAMPT on the skeleton may be multi‐dimensional.

Mitochondrial dysfunction and cell senescence are hallmarks of aging and are closely interconnected (Miwa et al., 2022). Mitochondrial fusion is a process whereby two neighbouring mitochondria tether and then fuse their outer membranes, followed by the fusion of the inner membranes. Mitochondrial fusion is an important means of rescuing compromised mitochondria to restore organelle health and homogeneity (Chen, Zhao, & Li, 2023). Since mitochondrial networks rely on fission and fusion, the loss of fusion and unbalanced fission events lead to a fragmented mitochondrial network, which in turn causes the cells to senescence (Harrington et al., 2023). A recent study showed that the depletion of *Nampt* disturbed mitochondrial homeostasis and causes neuronal degeneration in mouse hippocampus (Zhuan et al., 2022). NAMPT affects mitochondrial function by mediating the downstream effector FoxO3a (Zhuan et al., 2022). Dysfunction of mitochondrial fusion and decreased mitochondrial membrane potential in aging BMSCs were observed in this study. Overexpression of *Nampt* rescue mitochondrial fusion disorder in aging BMSCs, indicating that NAMPT exerts anti‐senescence effects by promoting mitochondrial fusion. The main components involved in mitochondrial fusion are members of the dynamin‐like family of GTPases‐Mitofusins 1 and 2 (MFN1/2) in the outer membrane and optic atrophy 1 (OPA1) in the inner membrane (Nyenhuis et al., 2023). We found that the expression of OPA1 was regulated by NAMPT. OPA1 expression was decreased in senescent cells. This study provides a preliminary description of the relationship between NAMPT, OPA1 and mitochondrial function. Further investigation is warranted to elucidate how NAMPT affects mitochondrial function and the expression or function of OPA1.

P7C3 is an aminopropyl carbazole reported as a neuroprotective compound in animal models of neurodegenerative diseases or nerve cell injury (Wang, Han, et al., 2014). P7C3 increases NAD^+^ synthesis from nicotinamide by activating NAMPT, a key rate‐limiting enzyme in the NAD^+^ salvage pathway (Wang, Han, et al., 2014). Our finding that P7C3 binds to NAMPT is consistent with previous studies (Wang, Han, et al., 2014). Our recent research has confirmed that P7C3 ameliorates bone loss by inhibiting osteoclast differentiation and promoting osteogenesis. Melanie Coathup et al. also confirmed that P7C3 acts as a countermeasure against ionizing radiation‐induced bone loss (Wei et al., 2023). The present study demonstrates P7C3 as a novel compound for the amelioration age‐related osteoporosis, inhibiting the senescence phenotype of BMSCs. Supplementation of senescence BMSCs with P7C3 significantly reduced adipogenic transcription factor expression and lipid droplet formation in vitro. Notably, a more than threefold increase in bone mineral deposition was found in the P7C3‐treated cells compared to the control. Our in vivo findings further support that senescent‐induced bone loss was rescued following P7C3 treatment. The anti‐senescence effect of P7C3 is closely related to the increase of NAMPT activity and OPA1 expression, promoting mitochondrial membrane fusion. Overall, based on P7C3 treatment and mechanism analysis, we identified beneficial molecular responses that attenuate bone loss and prevent SOP.

Some limitations of this study should also be mentioned. Firstly, our research demonstrates that NAMPT promotes osteogenic differentiation and inhibits adipogenic differentiation by delaying senescence of BMSCs, thus participating in bone metabolism. However, we did not investigate whether NAMPT affects osteoclast formation involved in bone metabolism. Mechanistically, we found that NAMPT increases SIRT3 expression, inhibits YME1L1 deacetylation, thereby suppressing OPA1 processing and promoting mitochondrial fusion. However, we did not further study the relationship between mitochondrial metabolism and osteoporosis in the aging process. Furthermore, we will investigate whether NAMPT directly affects osteogenic differentiation processes to regulate bone mass.

Controlling the senescence of BMSCs in favor of osteogenesis is considered a promising approach for treatment of osteoporosis. In summary, we demonstrate that NAMPT inhibits the senescence phenotype of BMSCs through by facilitating the level of OPA1 levels and mitochondrial fusion. Our findings highlight the importance of P7C3‐mediated NAMPT‐OPA1 pathway activation in ameliorating skeletal senescence phenotype. NAMPT activator P7C3 is a potential treatment strategy for SOP. Further work is needed to investigate the mechanism of NAMPT in senescent BMSCs and its regulation of mitochondrial function. This is conducive to find or construct more P7C3‐like compounds for the treatment of SOP.

## AUTHOR CONTRIBUTIONS

XZ, JB, HS conceptualised the study. All listed authors performed the experiments and the statistical analysis. CB, MZ, BT wrote original draft.

## FUNDING INFORMATION

This work was supported by the National Natural Science Foundation of China (No. 82472515, 82102611, 82402826); the Natural Science Foundation of Jiangsu Province (No. BK20210089); the Gusu health talent plan‐special talents C (2021) 021, (2022) 009; the Gusu health talent plan‐research project (GSWS2021015, GSWS2022038); China National Nuclear Corporation Young talent project; the Social Development Key Programs of Jiangsu Province‐Advanced Clinical Technology (BE2023705); Scientific research project of Jiangsu Provincial Health Commission (Z2022010); Nuclear Medicine Technology Innovation Project by China Nuclear Medical (ZHYLTD2023001); Clinical Innovation and Interdisciplinary Translation Project of Suzhou Medical College, Soochow University (ML12301523); Jiangsu Provincial Medical Key Discipline (Laboratory) Cultivation Unit (JSDW202223); Youth Excellence Talent Program of the Second Affiliated Hospital of Soochow University (XKTJ‐RC202406).

## CONFLICT OF INTEREST STATEMENT

Authors in this study declare no conflict of interest.

## Supporting information


Figure S1.

Figure S2.

Figure S3.

Figure S4.

Figure S5.

Table S1.

## Data Availability

All data needed to evaluate the conclusions in the paper are present in the paper and the Supplementary Materials.
