# Supplementary figures and images for: Targeting NAMPT‐OPA1 for treatment of senile osteoporosis
Source: Aging Cell. 2024 Nov 14;24(3):e14400. doi: 10.1111/acel.14400 (PMC11896342; doi:10.1111/acel.14400)

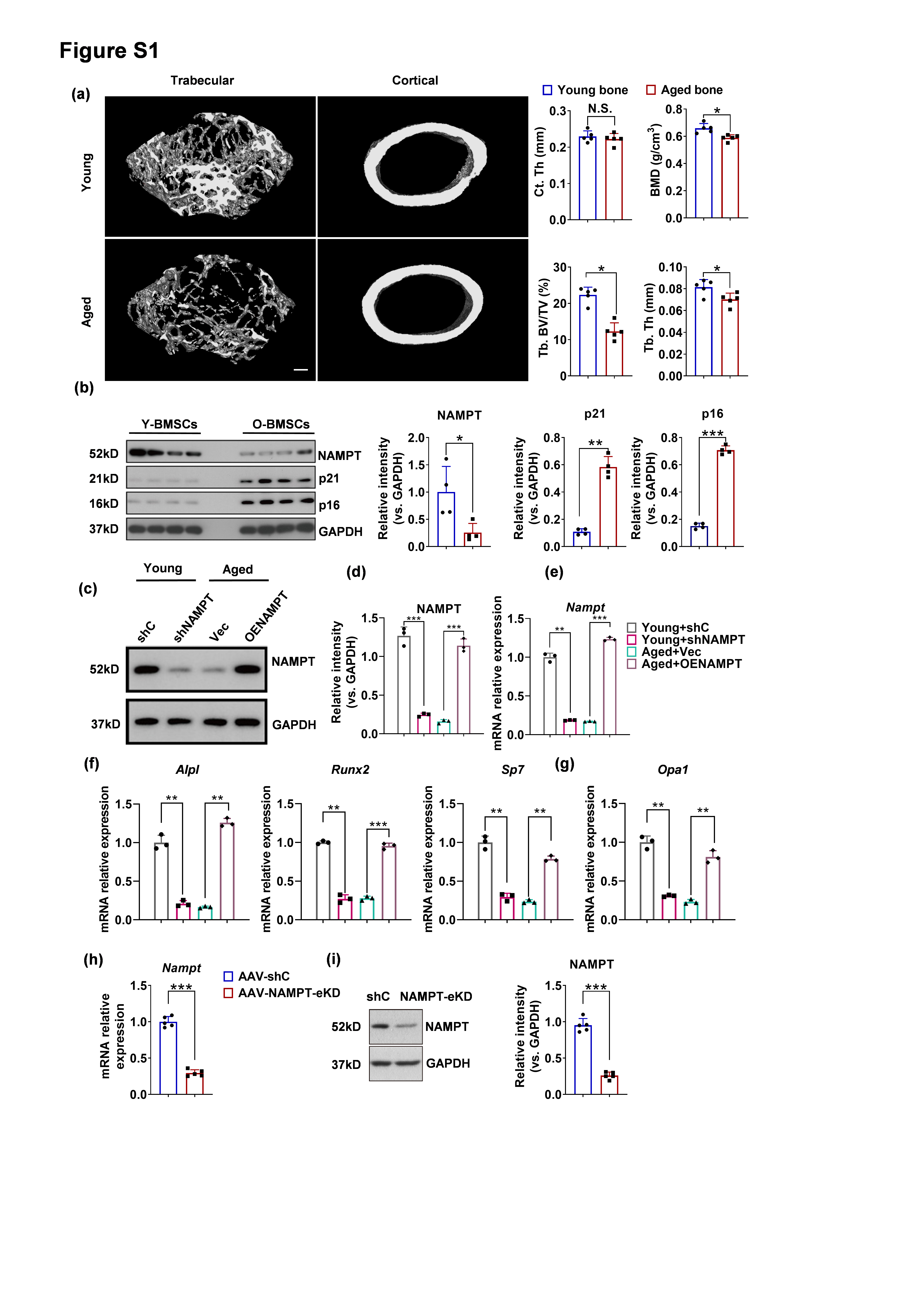

Supplement: Supplementary file 1 — Figure S1. [file ACEL-24-e14400-s002.tif]

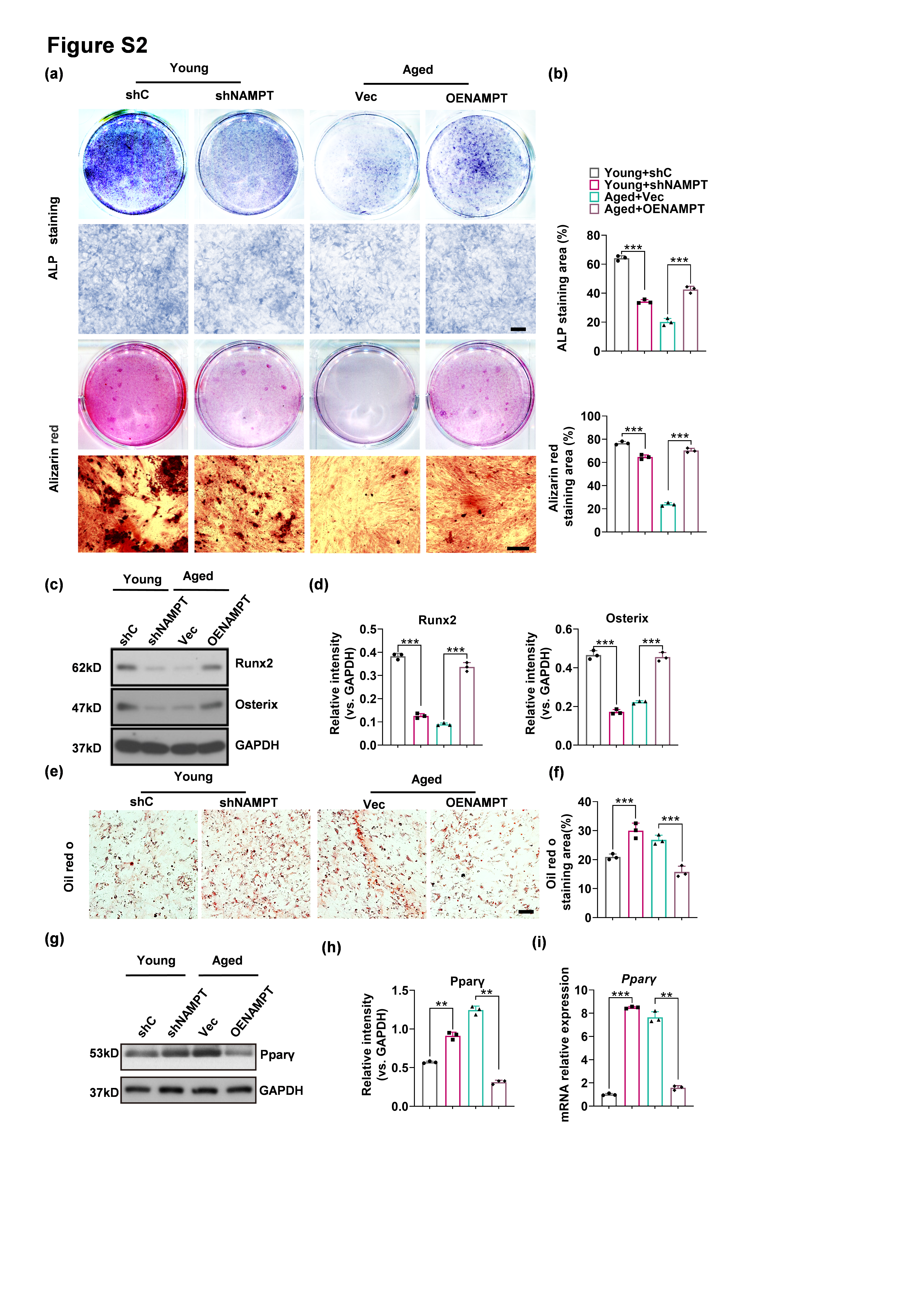

Supplement: Supplementary file 2 — Figure S2. [file ACEL-24-e14400-s004.tif]

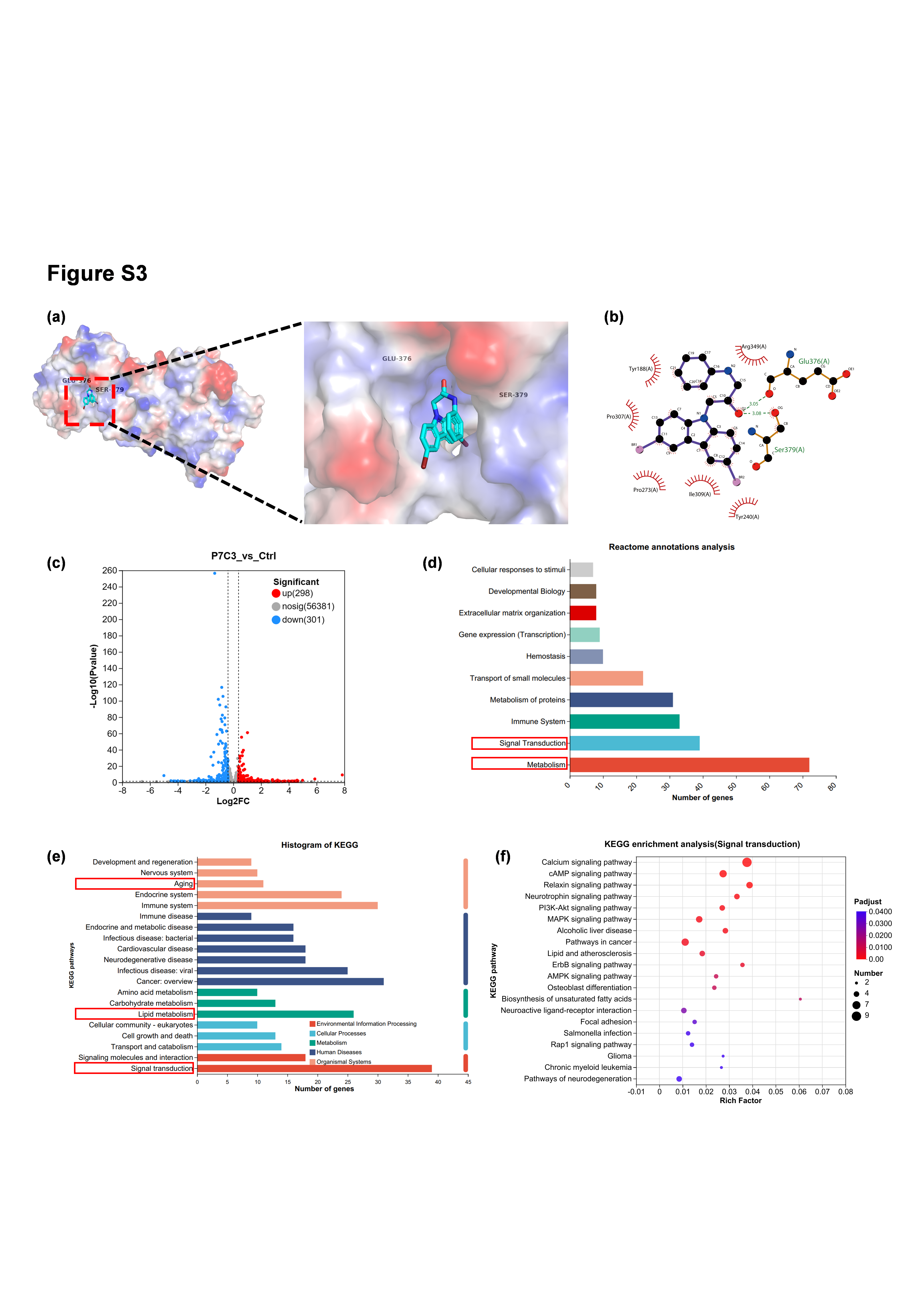

Supplement: Supplementary file 3 — Figure S3. [file ACEL-24-e14400-s006.tif]

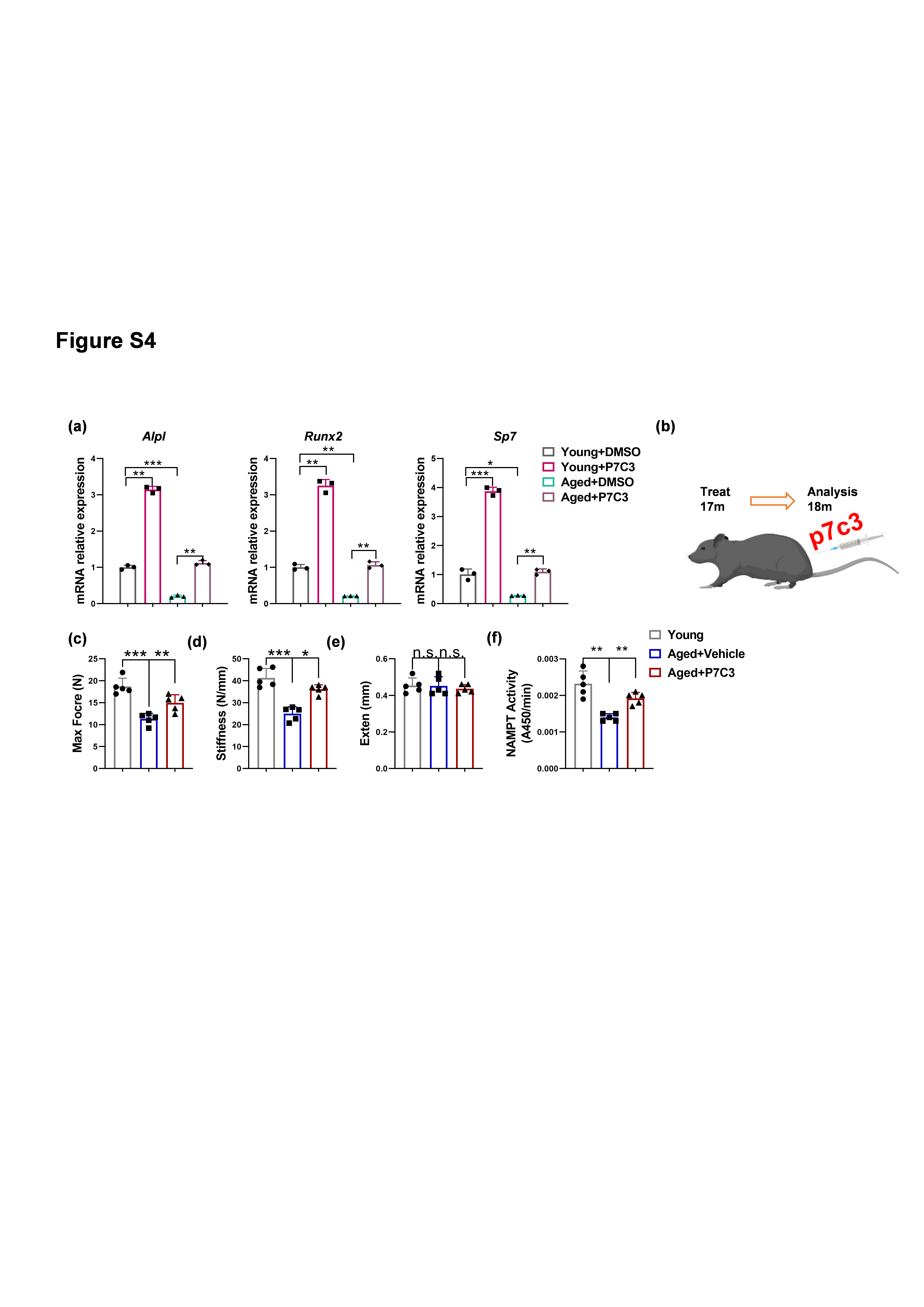

Supplement: Supplementary file 4 — Figure S4. [file ACEL-24-e14400-s005.tif]

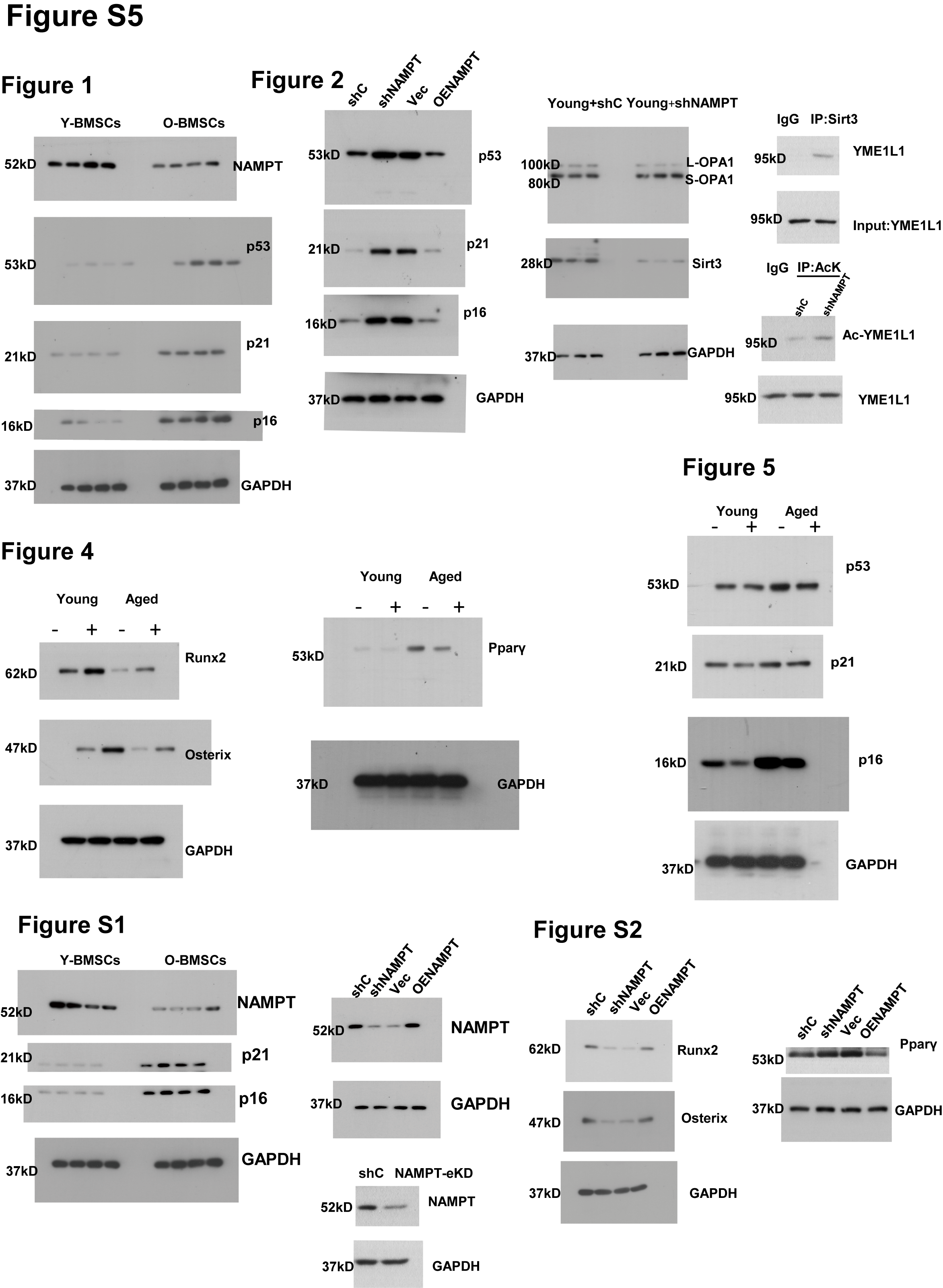

Supplement: Supplementary file 5 — Figure S5. [file ACEL-24-e14400-s001.tif]
