# Supplementary material for: Targeting NAMPT‐OPA1 for treatment of senile osteoporosis
Source: Aging Cell. 2024 Nov 14;24(3):e14400. doi: 10.1111/acel.14400 (PMC11896342; doi:10.1111/acel.14400)
Supplement: Supplementary file 6 — Table S1. [file ACEL-24-e14400-s003.docx]

Inclusion criteria for clinical patients:

Elderly patients diagnosed with osteoporosis and experienced fractures due to accidents.

Young patients with normal bone mass who experienced fractures due to accidents.

Exclusion criteria:

Elderly patients receiving anti-osteoporosis treatments such as PTH therapy.

Secondary osteoporosis due to systemic metabolic bone diseases (thyroid-related diseases, Cushing's syndrome, connective tissue diseases, etc.).

Use of medications (e.g., steroids) affecting bone metabolism

Basic patient information：

| Patient | Gender | Age | Bone mineral density (T-value) | Drug intake | Complicating disease |
| --- | --- | --- | --- | --- | --- |
| Y-1 | male | 26 | 1.0 | No | No |
| Y-2 | male | 32 | 1.2 | No | No |
| Y-3 | male | 33 | 1.5 | No | No |
| Y-4 | male | 39 | 2.1 | No | No |
| O-1 | male | 77 | -2.9 | Hypotensor | Hypertension |
| O-2 | male | 79 | -3.8 | Hypotensor | Hypertension |
| O-3 | male | 80 | -4.5 | No | No |
| O-4 | male | 81 | -3.6 | No | No |
